# LRRC6 regulates biogenesis of motile cilia by aiding FOXJ1 translocation into the nucleus

**DOI:** 10.1186/s12964-023-01135-y

**Published:** 2023-06-16

**Authors:** Dong Yun Kim, Yu Jin Sub, Hye-Youn Kim, Kyeong Jee Cho, Won Il Choi, Yo Jun Choi, Min Goo Lee, Friedhelm Hildebrandt, Heon Yung Gee

**Affiliations:** 1 Department of Pharmacology, Graduate School of Medical Science, Brain Korea 21 Project, Yonsei University College of Medicine, Seoul, 03722 Republic of Korea; 2 Division of Gastroenterology, Department of Internal Medicine, Severance Hospital, Seoul, Republic of Korea; 3 Division of Nephrology, Boston Children’s Hospital, Harvard Medical School, Boston, MA 02115 USA

**Keywords:** Motile cilia, Primary ciliary dyskinesia, LRRC6, FOXJ1, Nuclear translocation

## Abstract

**Background:**

LRRC6 is an assembly factor for dynein arms in the cytoplasm of motile ciliated cells, and when mutated, dynein arm components remained in the cytoplasm. Here, we demonstrate the role of LRRC6 in the active nuclear translocation of FOXJ1, a master regulator for cilia-associated gene transcription.

**Methods:**

We generated Lrrc6 knockout (KO) mice, and we investigated the role of LRRC6 on ciliopathy development by using proteomic, transcriptomic, and immunofluorescence analysis. Experiments on mouse basal cell organoids confirmed the biological relevance of our findings.

**Results:**

The absence of LRRC6 in multi-ciliated cells hinders the assembly of ODA and IDA components of cilia; in this study, we showed that the overall expression of proteins related to cilia decreased as well. Expression of cilia-related transcripts, specifically ODA and IDA components, dynein axonemal assembly factors, radial spokes, and central apparatus was lower in Lrrc6 KO mice than in wild-type mice. We demonstrated that FOXJ1 was present in the cytoplasm and translocated into the nucleus when LRRC6 was expressed and that this process was blocked by INI-43, an importin α inhibitor.

**Conclusions:**

Taken together, these results hinted at the LRRC6 transcriptional regulation of cilia-related genes via the nuclear translocation of FOXJ1.

Video Abstract

**Supplementary Information:**

The online version contains supplementary material available at 10.1186/s12964-023-01135-y.

## Background

Multiple motile cilia perform beating function in a single to produce a synchronized wave-like motion, and defects in these organelles cause disorders in developmental stages and in several organs [1–4]. During the developmental stage of the embryonic node, incomplete beating motion of motile cilia causes laterality defects, such as body asymmetry randomization and situs inversus totalis. Motile cilia are responsible for the flow of fluid, and improper function causes infertility by preventing sperms and eggs to localize properly in the reproductive tracts, as well as causing the accumulation of cerebrospinal fluid in the ventricles of the brain, leading to hydrocephalus [5, 6]. In addition, dysfunction in mucosal clearance in the respiratory system, which is one of the important functions of motile cilia, can increase the risk of respiratory infection [7, 8]. The aforementioned disorders are collectively defined as primary ciliary dyskinesia (PCD).

So far, approximately 57 genes have been identified as causing motile ciliopathies when mutated, accounting for 70% of cases. Most of these genes encode subunits of axonemal structures of motile cilia [3, 9]. The 9 + 2 microtubule structure of axoneme has one singlet pair in center, surrounded by nine microtubule doublets. Outer dynein arm (ODA) and inner dynein arm (IDA) are attached to the nine microtubule doublets at regular intervals, providing ATP-dependent motor activity. Accessory proteins, namely, radial spoke and nexin, connect central microtubule singlets to outer microtubule doublets and between outer microtubule doublets, contributing to the synchronized movement of motile cilia [10, 11].

ODA is a large multi-subunit protein complex comprising light, intermediate, and heavy chains [12]. ODA is known to be preassembled in a stepwise manner in the cytoplasm before being transported into motile cilia [13]. Cytoplasmic preassembly of ODA complex involves the folding of globular dynein head domains with heavy chains following the assembly of complex constituting heavy and intermediate chains. These processes are regulated and assisted by dynein axonemal assembly factors (DNAAFs), the defects of which result in PCD [9]. DNAAFs are localized to the cytoplasm of motile ciliated cells, and to date, 15 DNAAFs and 4 cilia- and flagellar-associated proteins have been identified [14]. Yet, the exact functions of these cytoplasmic proteins in the preassembly of ODA complex remain to be defined.

*LRRC6*, also known as Seahorse, was first identified as a candidate gene for cystic kidney phenotype in zebrafish and was found to be required for establishing left–right symmetry. LRRC6 encodes a protein that contains leucine-rich repeats (LRRs), an LRR cap domain, a coiled-coil region, and a CS-like domain near the C-terminus [15]. An *LRRC6*-deficient mouse model has been reported to exhibit PCD phenotypes in the absence of ODAs in the axon of motile cilia, but the previous reports focused only on the role of *LRRC6* as one of the DNAAFs [16]. Therefore, we aimed to reveal other potential pathological mechanisms by which the loss of LRRC6 leads to motile ciliopathy.

## Methods

### Mice

Targeted *Lrrc6*^tm1e(KOMP)Wtsi^ embryonic stem cells were obtained from the Knockout Mouse Project Repository and injected into blastocysts. Chimeric mice were bred with C57BL/6 J mice to establish germline transmission. Wild-type littermates were used as controls for *Lrrc6* knockout (KO) mice. Genotyping was performed by standard PCR using the following primers: CDS-neoF (5′- GGGATCTCATGCTGGAGTTCTTCG -3′), CDS-Lrrc6-F (5′- TCTGAATACTTCCCTGCTTTGTGGC -3′), and CSD-Lrrc6-R (5′- AAACTTCTACAGTCCCTCCACACCC -3′).

### β-Galactosidase staining

P14 mice were sacrificed, and the testes and lungs were dissected. After three washes with phosphate-buffered saline (PBS), the tissues were fixed in 4% paraformaldehyde (PFA)/0.02% Nonidet P-40 (NP-40) for 2 h at room temperature and permeabilized with 0.02% NP-40 in PBS for 1 h. Samples were incubated overnight at 37 °C in X-gal staining solution composed of 5 mM K_3_Fe(CN)_6_, 5 mM K_4_Fe(CN)_6_, 2 mM MgCl_2_, 0.01% sodium deoxycholate, 0.02% NP-40, and 1 mg/mL X-gal in PBS. They were then washed three times with PBS for 5 min each wash and post-fixed with 4% PFA for 24 h before being embedded in paraffin. Sections (10 μm thick) were deparaffinized and rehydrated through a graded series of ethanol concentrations followed by counterstaining with Nuclear Fast Red (Vector Laboratories, Burlingame, CA, USA).

### Histology

The brain and snout tissue specimens were fixed using 4% formalin for 24 h and decalcified with 500 mM ethylenediaminetetraacetic acid (EDTA) solution for 7 days. The brain and snout tissue specimens were cut into coronal sections with 5-μm thickness and stained with hematoxylin and eosin (H&E) and periodic acid-Schiff for histological examination.

### Transmission electron microscopy (TEM)

The tracheae of wild-type and *Lrrc6* KO mice at P10 were dissected and fixed using 2.5% glutaraldehyde, 1.25% PFA, and 0.03% picric acid in 0.1 M sodium cacodylate buffer (pH 7.4) overnight at 4 °C. Samples were then processed for TEM analysis using standard techniques.

### Assessment of ciliary basal feet orientation

Ciliary basal feet orientation was measured as previously described [17]. Using ImageJ software (National Institutes of Health, Bethesda, MD, USA), a line was drawn along the individual cilia and the angle of the line was measured. Measured angles of cilia were normalized by the average angle of all the cilia within the cell in order to eliminate possible differences. Polar plots for the distribution of ciliary angles were generated using ggplot2 in R. The statistical method, F-test, was used to assess the data and was calculated using the var.test() function in R.

### Imaging motility of cilia

For preparation of mouse brain ependymal cells, mouse were anesthetized and perfused using Isoflurane (Hana pharm, Gyeounggi) and PBS respectively. Brain is extracted from the mouse and placed in ice-cold PBS. The brain in PBS is cut into coronal sections with 100 μm using vibratome (Leica, Germany) and transferred into confocal plate (SPL Life Science; Pocheon, Korea, #100,351). For preparation of imaging organoids, basal oragnoids embedded in matrigel were laid on the confocal plate with transwell inserts. Motility of cilia was examined (100 frames/second) with a Axio Observer A1 (Carl Zeiss, Germany) and motion pattern of cilia was traced with NIS-Elements software (Nikon, Japan).

### Cell culture and transfection

HEK 293 T cells and HeLa cells were maintained in Dulbecco's Modified Eagle Medium (DMEM) supplemented with 10% fetal bovine serum (FBS) and penicillin (50 IU/mL) or streptomycin (50 μg/mL). The cells were transfected with plasmids using Lipofectamine PLUS reagent (Invitrogen; Carlsbad, CA, USA).

### Nuclear transport inhibitor treatment

To identify importins related to the nuclear translocation of FOXJ1, HeLa cells were transfected with a *Lrrc6* × *gfp* expression vector and/or a *Foxj1* expression vector using Lipofectamine 2000 (Invitrogen, CA, USA, #11,668–09). After 24 h of transfection, the HeLa cells were treated with either DMSO for 24 h, with INI-43 (Sigma-Aldrich, #SML1911) for 24 h, or with Ivermectin (Sigma-Aldrich, #i8898) for 24 h. After washing with PBS and fixation with 4% PFA, cells were observed under a fluorescence microscope.

### Antibodies

LRRC6 and DNAH5 antibodies were previously described [18, 19]. Antibodies against REPTIN (ab89942; Abcam, Cambridge, UK), IQUB (HPA020621; Sigma-Aldrich, MO, USA), TCTEX1D1 (HPA028420; Sigma-Aldrich, MO, USA), DYX1C1 (info. Omitted), C21ORF59 (sc-365792; Santa Cruz Biotechnology, TX, USA), DNAI1 (SAB4501181; Novus Biologicals, Littleton, CO, USA), DNAI2 (H00064446-M01; Abnova, Taipei, Taiwan), DNAH7 (NBP1-93,613; Novus Biologicals, Littleton, CO, USA), acetylated α-tubulin (T7451 from Sigma-Aldrich and 5335S from Cell Signaling Technology, Danvers, MA, USA), FLAG (#8146; Cell Signaling Technology, Danvers, MA, USA), Myc (#2276; Cell Signaling Technology, Danvers, MA, USA), FOXJ1 (sc-53139; Santa Cruz Biotechnology, TX, USA), KPNB1(nb100-79,806; Novus Biologicals, Littleton, CO, USA), and β-actin (sc-1615; Santa Cruz Biotechnology, TX, USA) were purchased from commercial sources. Secondary antibodies were purchased from Invitrogen and Santa Cruz Biotechnology for immunofluorescence and immunoblotting analyses.

### Immunofluorescence (IF) analysis

The tracheal tissue was fixed in 4% PFA overnight at 4 °C, embedded in a paraffin block, and cut into 5-μm-thick sections. The sections were then mounted on slides, deparaffinized, and rehydrated through a graded series of ethanol concentrations. After rehydration, antigen retrieval was performed by boiling the sections for 30 min using a Retrieve-All Antigen unmasking system 1 (pH 8; BioLegend, San Diego, CA, USA). Sections were permeabilized with 1% sodium dodecyl sulfate for 10 min at room temperature. The tracheal sample was incubated in blocking buffer containing 10% donkey serum and 1% bovine serum albumin (BSA) for 1 h at room temperature. Samples were incubated overnight at 4 °C with primary antibodies diluted in the blocking buffer. After washing the samples with PBS, they were incubated with secondary antibodies diluted in the blocking buffer for 30 min at room temperature and then washed with PBS before mounting on slides using a mounting medium and cover slips. Images were acquired using the LSM 700 microscope (Carl Zeiss; Jena, Germany).

### Tandem mass tag–mass spectroscopy (TMT-MS)

An 11-plex pool of TMT labeled peptide samples was prepared as previously described, with a slight modification to the method [20]. Briefly, five wild-type and six *Lrrc6* KO mouse testes were homogenized using 8 M urea, then reduced using tris(2-carboxyethyl)phosphine hydrochloride (TCEP), and alkylated using iodoacetamide (IAA). The proteins were centrifuged at 21,000 g to remove pellets. After the buffer of the supernatants was exchanged, protein concentration was quantified by measuring the absorbance at 205 nm on the NanoDrop™ 2000c spectrophotometer (Thermo Fisher Scientific, MA, USA) and adjusted to 2.2 μg/μL. The protein samples were digested with trypsin at 37 °C overnight at 1:50 trypsin ratio. The tryptic peptide samples were labeled with TMT11 based on a TMT/protein ratio (w/w) of 1:1 for 30 min, and an equal amount of each labeled sample was pooled. The pooled peptide samples of 11-plex were fractionated via basic reversed-phase liquid chromatography using a 50-cm C18 capillary column. The peptides were eluted in a 52-min gradient, and 42 fractions were collected every minute and concatenated into 10 fractions. Peptides were detected using Orbitrap Fusion Lumos, as previously described [21]. The raw-files were imported to IP2 pipeline, which searched the generated MS data against the UniProt *Mus musculus* database downloaded from the European Bioinformatics Institute website (https://www.uniprot.org/proteomes/UP000000589) and filtered the data using the default parameters [22].

### RNA sequencing

Total RNA was isolated from the testicular tissues (P21) obtained from wild-type and *Lrrc6* KO mice using a Qiagen RNA extraction kit (Qiagen; Valencia, CA, USA). RNA sequencing was performed by Theragen Etex (Suwon, Korea). Libraries were constructed using a TruSeq RNA Library Sample Prep kit (Illumina; San Diego, CA, USA), and the enriched library was sequenced on an Illumina HiSeq 2500 system. CLC Genomics Workbench 9.5.3 software (Quiagen, Germany) was used to map the reads to the mouse genome (NCBI GRCm38/mm10).

### Transcriptomic and statistical analysis

All bioinformatic analyses were performed on R v4.1.2. Further analyses of TMT-MS data, such as protein annotation, data normalization, and identification of differentially expressing proteins (DEPs) was performed with the R package Proteus (version 0.2.15). Gene set enrichment analysis was performed using the R package MSigDBr (version 7.5.1) with the false discovery rate (FDR) set at 0.05 to identify the significantly enriched gene set. Hierarchical clustering of RNA-seq data was performed using the R package Pheatmap (version 1.0.12). Differentially expressing genes (DEGs) were identified using the R package DESeq2 (version 1.34.0). DEGs were selected based on absolute fold changes of more than 2 and *p*-values of less than 0.05, as measured in Gaussian *T*-test. Gene ontology (GO) analysis of DEGs was performed using the R package GProfiler2 (version 0.2.1) and GO terms with *p*-value less than 0.05 (Benjamini-Hochberg-corrected) were considered statistically significant. Statistical results are presented as means ± standard errors or standard deviations for the indicated number of experiments. Continuous data was statistically analyzed using two-tailed Student’s *t*-test or one-way analysis of variance, with Dunnet’s, Bonferroni, or Dunn post hoc test conducted as appropriate. Results with *p*-values of less than 0.05 were considered statistically significant.

### Basal organoid culture and whole mount

Basal stem cells were isolated from mice at P14 following a method previously described [23], with a slight modification. Briefly, basal stem cells were dissociated from tracheal tissue through a 30-min digestion with Dispase (16U/mL; BD Biosciences, San Jose, CA, USA) at 37 °C. The digestion reaction was quenched by treating the trachea with 10% FBS in PBS (PF10), and the epithelium including basal stem cells were peeled out by passing 5 mL of PF10 through the trachea. After a quick wash with 1 mL of cold PBS, the epithelium was further dissociated into single cells using TrypLE Express buffer (Thermo Fisher Scientific, MA, USA) at 37 °C for 10 min in a shaking incubator. Digestion was quenched with PF10, followed by a quick wash with PBS. Cells were resuspended in mTEC/Plus media, mixed 1:1 with growth factor–reduced Matrigel (BD Biosciences, San Jose, CA, USA), and 100 μL pipetted into a 24-well 0.4-μm Transwell® insert (SPL Life Science; Pocheon, Korea). For the proliferation of stem cells and differentiation of basal organoid, mTEC/Plus and mTEC/SF medium were sequentially applied to the basal chambers of the wells for 7 consecutive days. Cells were cultured at 37 °C in 5% CO_2_. For whole mount of basal organoid, fully differentiated organoids were gently dissociated from Matrigel using cell recovery solution (Corning, New York, USA) at 4 °C for 45 min. Basal organoids were fixed with 4% formalin for 30 min in microcentrifuge tubes and permeabilized with 0.3% Triton X-100 for 20 min at room temperature. The basal organoids were incubated in blocking buffer containing 10% donkey serum, 1% BSA, and 0.3% Triton X-100 for 1 h at room temperature. The samples were then processed for IF assays using standard techniques.

## Results

### Lrrc6 knockout resulted in PCD in mice

To elucidate the function of LRRC6, the embryonic stem cells containing the gene-trap cassette in the intronic region downstream of *Lrrc6* exon 3 (*Lrrc6*^tm1e(KOMP)Wtsi^) were microinjected into mice and founders were bred (Additional file 1: Fig. S1A). Allele-specific primers were used to genotype the mice (Additional file 1: Fig. S1A, B). The targeting cassette contained lacZ, and β-galactosidase staining showed that LRRC6 was expressed in the bronchi and bronchioles of the lung as well as in spermatids and earlier-stage germ cells of testis (Additional file 1: Fig. S1C–F).

*Lrrc6* KO mice were born at expected Mendelian ratios, indicating that the *Lrrc6* targeting allele does not cause embryonic lethality. However, *Lrrc6* KO mice grew slower and were notably smaller than wild-type (WT) mice at P10 (Additional file 1: Fig. S1G, H). *Lrrc6* KO mice died within one month of birth, and the mean survival was 14 days, reducing Mendelian ratio at weaning age (Additional file 1: Fig. S1I). Contrary to *Lccr6* KO mice, *Lrrc6* heterozygous mice were haplosufficient and survived normally, similar to WT mice. (Additional file 1: Fig. S1H, I).

**Fig. 1:**
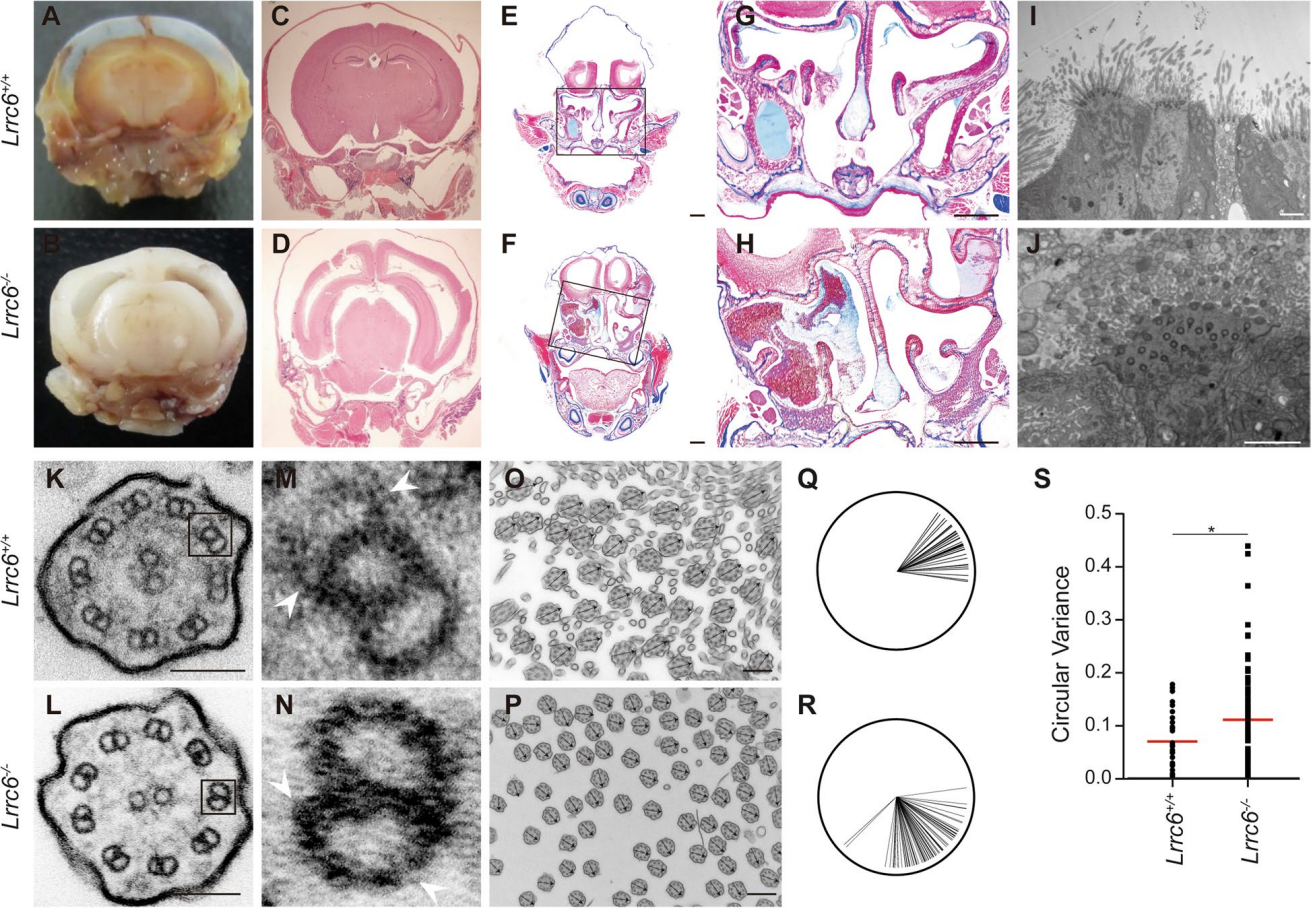
Absence of dynein arms and orientation polarity loss of central pairs in motile cilia axon underlie primary ciliary dyskinesia (PCD) phenotypes of *Lrrc6* KO mouse. **A-D** *Lrrc6* knockout (KO) mice exhibited hydrocephalus which is a characteristic head deformation in PCD. **C-D** Coronal sections of decalcified head of (**A**) wild-type (WT) and (**B**) *Lrrc6* KO mouse and hematoxylin and eosin (H&E) staining of the coronal brain sections of (**C**) WT and (**D**) *Lrrc6* KO mouse showed enlarged ventricular cavities and reduced cortical thickness in *Lrrc6* KO. Scale bar, 1 mm. **E–H** Nasal cavities of *Lrrc6* KO mice showed a loss of mucociliary clearance ability. H&E staining of the paranasal cavities of (**E**) WT and (**F**) *Lrrc6* KO mice showed mucus congestion along the nasal epithelium in *Lrrc6* KO mice. Scale bar, 1 mm. **H** Magnified mucus-accumulated region in *Lrrc6* KO mice and (**G**) the corresponding region in WT mice. Scale bar, 500 μm. **I-S** Transmission electron microscopy (TEM) analysis was conducted on trachea tissue of WT and *Lrrc6* KO mice at postnatal day 14. Accumulation of mucus is scarcely visible in the TEM image of (**I**) WT (**J**) but is exclusively apparent in *Lrrc6* KO mice. Scale bar, 5 μm. (**K-L**) 9 + 2 microtubule structures of motile cilia were normal in both WT and *Lrrc6* KO mice. Scale bar, 100 nm. **M–N** Magnified outer microtubule doublet TEM images show the absence of inner and outer dynein arms (IDA/ODA) in *Lrrc6* KO mice. **O-S** Polarity of central microtubule singlet pairs orientation was lost in *Lrrc6* KO mice. Low-magnification cross-sectioned TEM images and polar plots displaying the distribution and orientation of central microtubule singlet pairs of motile cilia in (**O**, **Q**) WT and (**P**, **R**) *Lrrc6* KO mice. Scale bar, 500 nm. **S** Angular variances in polar plots were quantified and are shown as the dynamite plot with mean ± SEM. *p < *0.05*

*Lrrc6* KO mice developed a predominantly evident hydrocephalus with complete penetrance (Fig. 1A–D, Additional file 1: Fig. S1J, K). Ventricles were extensively dilated, and the cerebral cortex became thin, which indicated an abnormal flow of cerebrospinal fluid (Fig. 1A–D). Nasal cavity of *Lrrc6* KO mice was filled with mucus, showing a defect in mucociliary clearance [24] (Fig. 1E–H); 47% of *Lrrc6* KO mice expressed laterality defects, including the reversal of the heart apex, stomach, liver, or spleen, implying insufficient cell movement during the prenatal developmental stage (Additional file 1: Fig. S1I–O). The aforementioned traits suggest that *Lrrc6* KO mice model human PCD [2, 3, 9, 25–28].

### Motile cilia structure in Lrrc6 KO mice hinted at an attenuation of synchronized motility

Considering that the main causes of PCD are motile cilia defects, we examined the ultrastructure of motile cilia using transmission electron microscopy (TEM) in mouse trachea. Unlike that of WT mice, tracheal epithelium of *Lrrc6* KO mice was covered with debris and mucus, consistent with the result of H&E staining that showed a mucus-filled nasal cavity in the *Lrrc6* KO mice (Fig. 1I, J, Additional file 1: Fig. S1P-Q). However, based on the microscopy results of longitudinal sections along the cilia of WT and *Lrrc6* KO mice, not only were the differences of the number, length, and width of cilia not significant, the basal bodies did not appear to differ from each other as well (Additional file 1: Fig. S1R-S). The typical 9 + 2 microtubule structure of motile cilia was also intact in *Lrrc6* KO mice (Fig. 1K, L).

To investigate the reason for the PCD phenotype in *Lrrc6* KO mice, we scaled up the magnification in the cross-section microscopy and found that outer and inner dynein arm (ODA/IDA) protein complexes, generally attached to both sides of outer microtubule doublets in motile cilia, were partially absent in *Lrrc6* KO mice (Fig. 1M, N). The ODAs and IDAs are force-producing molecular motors that cause the doublet microtubules to slide with respect to one another [29]. Not all ODAs and IDAs were absent, but partial loss of ODAs or IDAs appeared to be sufficient to interfere with the motility of cilium [30, 31].

While observing the cross-sections closely, we noticed that the central pairs of microtubules residing in the motile cilia of *Lrrc6* KO mice were orientated in a random direction, as opposed to the parallel direction seen in the orientation of motile cilia in WT mice (Fig. 1O–R, Additional file 1: Fig. S1T-U). As uniform basal feet orientation plays a crucial role in the synchronized motility of motile cilia, we measured and analyzed the angles of basal feet orientation in our samples [17]. While more than 80% of the motile cilia in WT mice exhibited basal feet orientation within 20° (-10° ~ 10°) of each other, the basal feet orientation of motile cilia in *Lrrc6* KO mice was evenly distributed across all angles (Fig. 1Q–S, Additional file 2: Table S1). To confirm the attenuation of motility in *Lrrc6* KO mouse, we prepared the brain ependymal cells in ventricles and examined motility of motile cilia. As expected, WT motile cilia had beating motion, yet that of *Lrrc6* KO hardly moved (Additional file 3: Video S1 and Additional file 4: Video S2). The ultrastructure and the motility of the motile cilia suggested that the PCD phenotype in *Lrrc6* KO mice results from the disruption of two central aspects in motile cilia: partial ODAs and IDAs of outer microtubule doublets, and basal feet orientation of central microtubule pairs.

### The level of proteins conferring cilia motility decreased in Lrrc6 mice

**Fig. 2:**
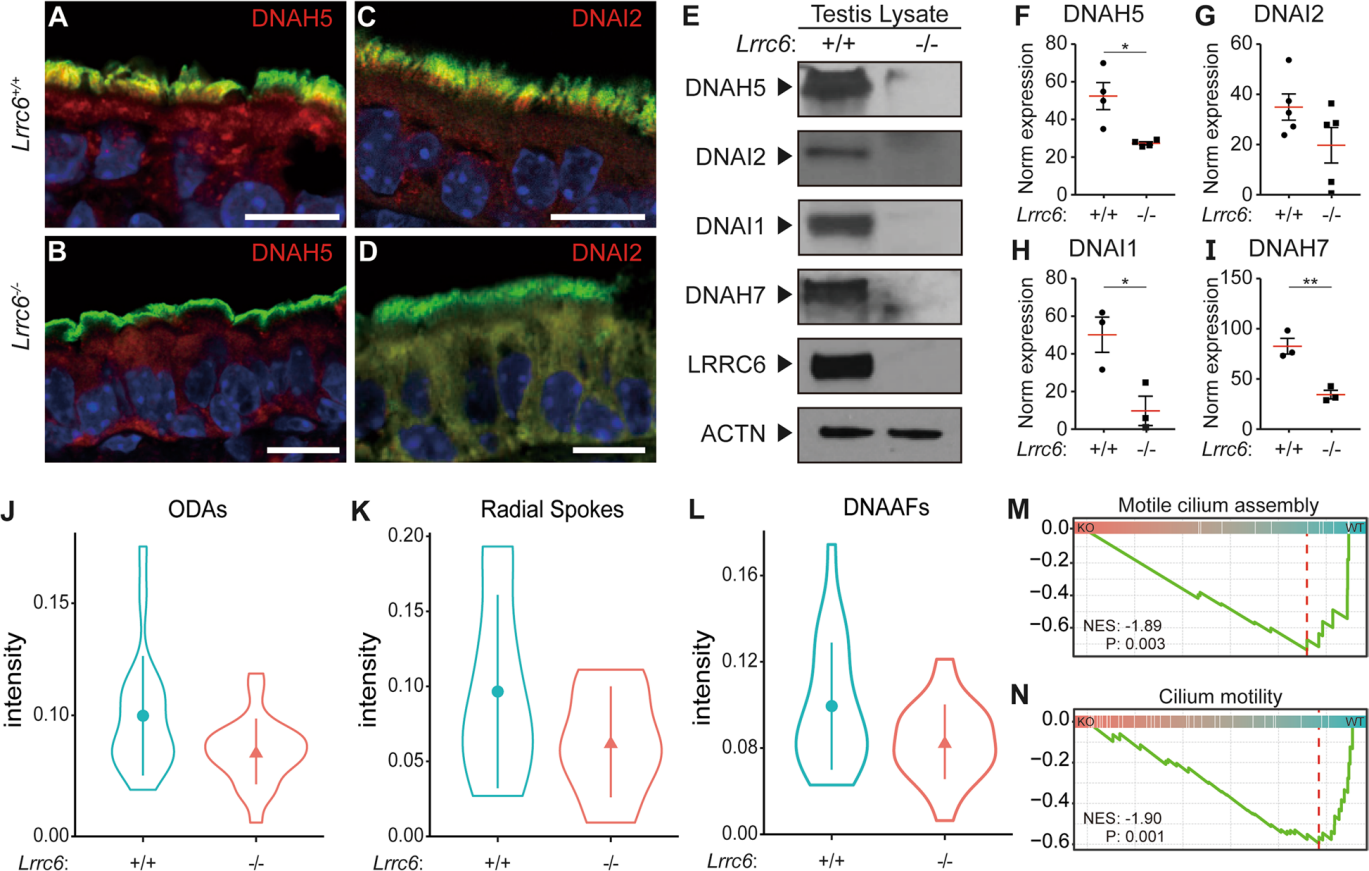
Reduced apparatus for synchronized ciliary motility in *Lrrc6* KO mice. **A-D** Immunofluorescence (IF) analysis of tracheal multi-ciliate epithelium showed a loss of outer dynein arm (ODA) subunits in *Lrrc6* KO mice. Red, (**A**, **B**) *DNAH5* and (**C**, **D**) *DNAI2*; green, acetylated α tubulin; blue, DAPI. **E-I** Overall protein expression level of ODAs was lower in the testicular extracts from *Lrrc6* KO than that in WT mice. I Representative immunoblot analyses of *DNAH5*, *DNAI2*, *DNAI1*, *DNAH7*, *LRRC6* and *ACTIN*. **F-I** Jitter plots represent band intensities normalized to *ACTIN* of the blot shown in panel (**E**), with the data points representing the mean ± SEM of more than three independent experiments against (**F**) DNAH5, (**G**) DNAI2, (**H**) DNAI1, and (**I**) DNAH7 respectively. * p < *0.05*; ** p < *0.01*. **J-N** Tandem mass tag mass spectroscopy (TMT-MS) analysis exhibited a decrease of protein expression of motile cilia accessory components. **J-L** Violin plots represent normalized intensities of (**J**) ODAs, (**K**) radial spokes, and (**L**) dynein arm assembly factors. **M–N** Enrichment plots represent protein expressions of genes classified under these specific GO terms; (**M**) motile cilia assembly and (**N**) cilium motility GO terms were enriched in WT mice compared to those in *Lrrc6* KO mice

Ciliated cells of PCD patients with *Lrrc6* mutation exhibited a cytoplasmic mislocalization of ODA subunit components [16]. Based on this finding, LRRC6 has been shown to function as a factor in mediating the interaction of ODA components with Zmynd10 for their preassembly in the cytoplasm and their transport into cilium axon [16, 32]. From the immunofluorescence assay results, however, contrary to the prediction that ODA components would be sequestrated from cilium but reside in cytoplasm, DNAH5 and DNAI2, two of the ODA components, were absent in the cytoplasm (Fig. 2A–D). To verify the decrease in the protein level of ODA and IDA components, western blot analysis on proteins isolated from mouse testis was conducted using antibodies against ODA components (DNAH5, DNAI1, and DNAI2) and an IDA protein (DNAH7). The result showed that all four protein levels were downregulated in *Lrrc6* KO mice (Fig. 2E–I).

To quantify the overall expression of motile cilia–related proteins accurately, proteins in mouse testis were analyzed on 11-plex tandem mass tag mass spectrometry (TMT-MS) (Additional file 1: Fig. S2A). An average of more than 50,000 peptides and more than 5,000 protein groups were identified per sample (Additional file 1: Fig. S2B). After normalization, differentially expressed proteins (DEPs) were identified, and a general decrease in ODA subunits along with dynein arm assembly factors and radial spoke–related proteins was noted, except for some undetected subunits (Additional file 1: Fig. S2C, Fig. 2J–L). Gene set enrichment analysis (GSEA) also revealed that proteins involved with motile cilium assembly and cilium- or flagellum-dependent cell motility were significantly downregulated in *Lrrc6* KO mice (Fig. 2M, N). Based on these results, we speculate that the function of LRRC6 is not merely limited to the post-translational regulation and interaction with chaperone and dynein arm subunit components but extends to the regulation of the whole ciliary machinery as well.

### Transcriptomic data correlated with protein expression pattern

**Fig. 3:**
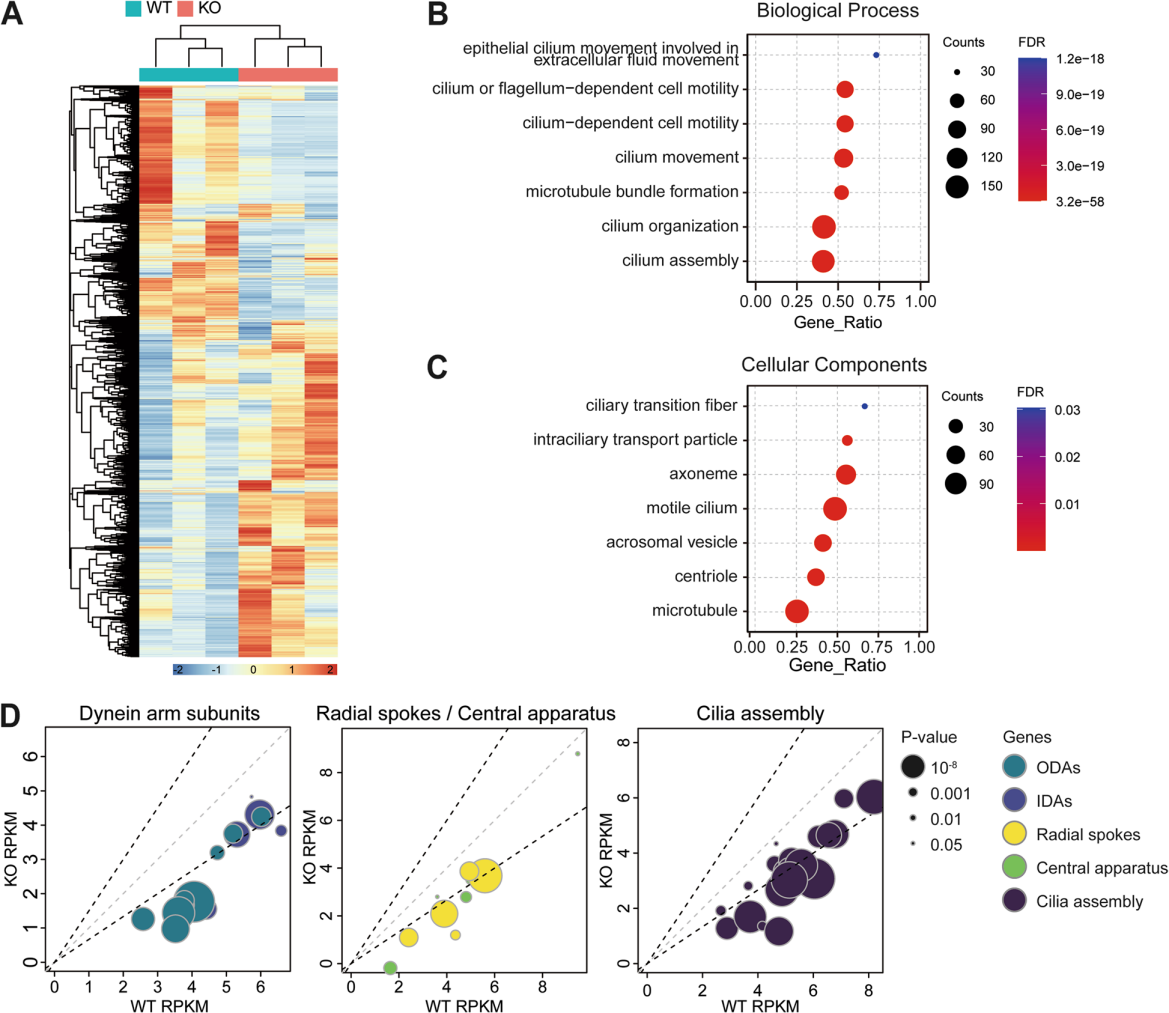
Transcription levels of cilia-related genes were downregulated. **A** The heatmap represent 641 differential expression genes (DEGs) between *Lrrc6* KO and WT mice. The DEGs include 56 upregulated genes and 585 downregulated genes. **B-C** Gene ontology (GO) evaluation indicated a major effect of downregulation in cilia-related genes in *Lrrc6* KO mice, particularly involving (**B**) biological processes including cilium assembly or cilium movement and (**C**) cellular components related to cilia. **D** Plots represent transcript expression levels of cilia-related genes. Sizes and colors of dots indicate *p*-value and function of the gene, respectively. Gray dashed lines, identical transcription level between WT and *Lrrc6* KO mice; left upper black dashed lines, transcription level of *Lrrc6* KO mice is 1.5-fold higher of WT mice; right lower black dashed lines, transcription level of WT mice is 1.5-fold higher of *Lrrc6* KO mice

To pinpoint the exact timing at which LRRC6 exerts its regulatory influence, transcriptome analysis was performed on mouse testicular RNA extracts. The overall quality of mRNA and sequencing data was acceptable, with no outliers after normalization (Additional file 1: Fig. S3A, B). It is worth noting that principal components analysis (PCA) plot on the transcriptomes of *Lrrc6* KO and WT mice revealed that knocking out *Lrrc6* had a widespread effect on gene transcription (Additional file 1: Fig. S3C). Differentially expressed genes (DEGs) that contributed to the transcriptomic differences between two groups were identified (Fig. 3A), and a gene ontology (GO) functional analysis was performed. GO analysis demonstrated that downregulated DEGs in *Lrrc6* KO were associated with functions subsumed under the biological process (BP) category, specifically with cilium assembly and motility. Additionally, the DEGs were also functionally related to cellular components (CC), particularly motile cilium and specific parts of cilium (Fig. 3B). In addition to the BP and CC functional categories, the enrichment of 47 cilia-related GO terms were statistically significant (not fully listed) (Additional file 1: Fig. S3D). Without a doubt, GSEA has validated a general decrease in transcript expression of genes involved in motile cilium assembly and cilium- or flagellum-dependent cell motor movement (Additional file 1: Fig. S3E).

Cilia-related proteins were divided into several groups by their role and location, thus the components of dynein arms, radial spokes, central apparatus, and a set of proteins taking part in cilia assembly were examined in a comparison analysis [33]. Practically all transcripts of genes belonging to the aforementioned groups decreased significantly (Additional file 1: Fig. S3F). More specifically, six, three, and 17 genes belonging to the latter three groups of proteins, respectively, were significantly decreased (Fig. 3D). As for the ODA and IDA components, the transcripts of 15 genes belonging to the protein group were decreased in the *Lrrc6* KO mice, with the decrease of seven of them being statistically significant (Fig. 3D).

### LRRC6 supported nuclear translocation of FOXJ1 by mediating interaction of FOXJ1 and Importin β_s_

LRRC6 has been hypothesized to translocate into the nucleus temporarily during the differentiation of ciliated cell, as it localizes to the cytoplasm of differentiated ciliated cells [34]. We have preliminarily demonstrated that wild-type mouse basal stem cells differentiated into multi-ciliated organoids when cultured in a tracheosphere culture assay (Additional file 1: Fig. S4A, B) [23]. The motile cilia of the basal organoids fully reproduced the motility of motile cilia in the mouse brain ependymal cells. Only the motile cilia of WT basal organoids showed beating motility, and that of *Lrrc6* KO was immotile (Additional file 5: Video S3 and Additional file 6: Video S4). Next, it was equally necessary to examine whether the intracellular location of protein in the organoid identically replicates its location in living tracheal tissues. Immunofluorescence assay against *DNAI2* and *DNAH5* was performed on the organoid and confirmed that the organoid effectively models the intracellular location of protein (Additional file 1: Fig. S4C). The differentiation process was followed up at 2-day interval from the time of serum starvation to the end of differentiation, yet the presence of LRRC6 was not observed in the nucleus (Additional file 1: Fig. S4D). As LRRC6 was not present in the nucleus at any point in time during the differentiation process and the transcripts of the cilia-related genes were low when *LRRC6* was not present, we speculated that LRRC6 likely did not modulate transcription levels directly.

**Fig. 4:**
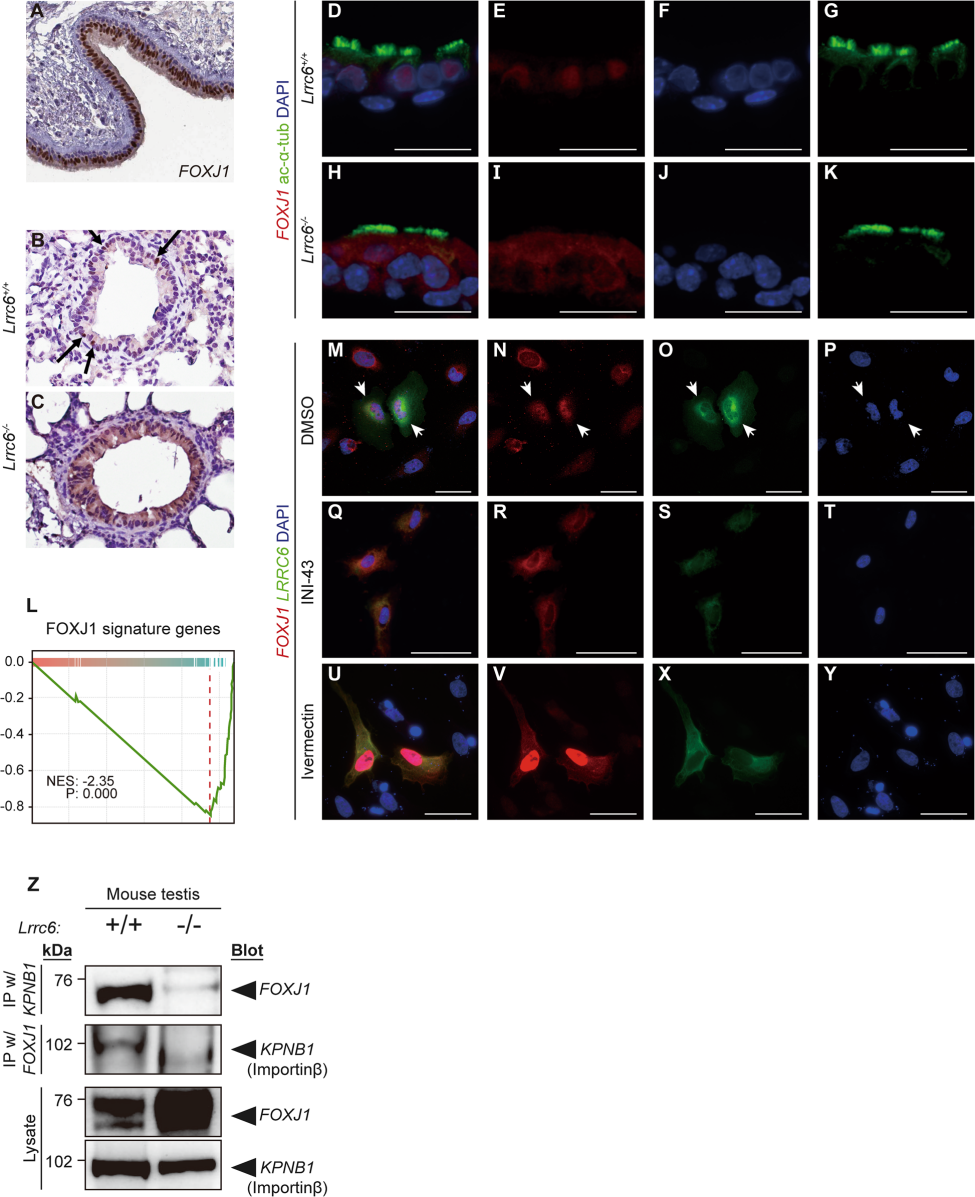
The presence of LRRC6 promotes FOXJ1 to translocate into nucleus and function as transcription factor. **A** The Human Protein Atlas database suggested that FOXJ1 protein resides in the nucleus of bronchus. Image of immunohistochemistry (IHC) against FOXJ1 in a sample from Patient id. T-26000; female; age 61. **B-C** Images of IHC against FOXJ1 in the lung alveoli of wild type (WT) and *Lrrc6* knockout (KO) mice. FOXJ1 is localized (**B**) specifically to the nuclei of WT alveolar cells (**C**) but remains in the cytoplasm of *Lrrc6* KO alveolar cells. **D-K** Images of immunofluorescence (IF) against FOXJ1 in the basal organoids of WT and *Lrrc6* KO mice. Scale bar, 50 nm. **I** Enrichment plot illustrates that the genes, whose transcriptional patterns were associated with the transcription of FOXJ1*,* were enriched in the testes of WT mice, not in the testes of *Lrrc6* KO mice. **M-P** IF assay against LRRC6 and FOXJ1 in cells with and without LRRC6 expression. White arrows mark two cells co-transfected with LRRC6 and FOXJ1 among the seven HeLa cells, and only FOXJ1 was transfected to the other cells. In the cells marked by the white arrows, FOXJ1 is predominantly visible in the nucleus rather than in the cytoplasm. Scale bar, 50 nm. **Q-T** Images of IF against LRRC6 and FOXJ1 in co-transfected HeLa cells under different drug-treated conditions show that INI-43 blocked translocation of FOXJ1 into the nucleus and that Ivermectin failed to inhibit translocation of FOXJ1. Scale bar, 50 nm. **Z** Immunoprecipitation (IP) results using importin β (KPNB1) and FOXJ1 showed that both proteins were co-precipitated only in wild-type mouse testes

To explore this further, the relationship of LRRC6 with other master transcription factors of ciliogenesis was assessed. Forkhead transcription factor 1 (FOXJ1), also termed forkhead homologue 4, is a critical regulator in the maintenance of airway epithelial cell differentiation through the regulation of ciliogenesis [35, 36]. In Human Protein Atlas database, *FOXJ1* was confirmed to be located inside the nucleus of human tracheal epithelial cells (Fig. 4A). We verified this in our study and observed FOXJ1 localizing to the nucleus of multi-ciliated cells in the trachea of WT mice, but FOXJ1 was also predominantly present in the cytoplasm of multi-ciliated cells in *Lrrc6* KO mouse trachea, though some was still apparent in the nucleus (Fig. 4B, C). The location of FOXJ1 in the basal cell organoid was also compared on the basis of *Lrrc6* genotype to determine whether its intracellular location was influenced by the presence of *LRRC6*. In wild-type basal cell organoid, the nuclear compartment was FOXJ1-positive (Fig. 4D–G). In contrast, FOXJ1 was detected in the cytoplasm instead of the nucleus in the *Lrrc6* KO organoid (Fig. 4H–K). As *FOXJ1* is a transcription factor, we confirmed that the expression level of FOXJ1 signature genes was greatly reduced in the associated transcriptomic data [33] (Fig. 4L). To verify that the apparent discrepancy in the intracellular location of FOXJ1 was not an unrelated side effect of *Lrrc6* gene ablation, LRRC6–green fluorescent protein (GFP) expression vector and FOXJ1 protein–expressing plasmid were co-transfected into HeLa cells. When immunofluorescence was performed, the FOXJ1 protein signal in the nucleus was much more intense in cells co-transfected with *Lrrc6* than in cells transfected solely with *Foxj1*-expressing plasmid. (Fig. 4M–P).

The presence of a monopartite nuclear localization signal (NLS) sequence, a type of classic NLS (cNLS), has been reported in FOXJ1, specifically across its 206–224 amino acid residues. The combinations of importins required for protein nuclear translocation differ depending on the type of NLS sequence in a particular protein, and proteins with cNLS require importin α and β [37]. When the co-transfected HeLa cells were treated with inhibitors of importin α and importin β, INI-43 and Ivermectin, respectively, we anticipated that FOXJ1 in the co-transfected cells and in the controls would localize to the cytoplasm. Instead, in the Ivermectin-treated cells, FOXJ1 remained in the nucleus while it localized to the cytoplasm in the INI-43-treated cells (Fig. 4Q–Y). These results are indicative of the predominant process by which FOXJ1 translocate into the nucleus, which entails an interaction solely with importin β, and they imply that FOXJ1 possesses a nonclassical NLS. Potential NLS sequences other than known classical NLS sequences were queried based on the amino acid sequences of FOXJ1 and it was confirmed that hydrophobic PY-NLS (hPY-NLS), a type of nonclassical NLS (ncNLS), was present across the amino acid residues 71–125 in FOXJ1 [38] (Additional file 1: Fig. S5A).

To determine whether the presence of LRRC6 affects the functional interaction between FOXJ1 and importin β, we performed an immunoprecipitation analysis using testis lysates from Lrrc6 wild-type and KO mice. Our results showed that FOXJ1 and importin β co-precipitated with each other in the tissue of wild type mice, but not in the tissue of Lrrc6 KO mice (Fig. 4Z). In conclusion, our findings suggest that LRRC6 facilitates the nuclear translocation of FOXJ1 by enhancing the physical interaction between FOXJ1 and importin β, thus facilitating the transcriptional cascade of ciliogenesis.

## Discussion

PCD is a rare autosomal recessive disorder that is characterized by dysfunctional mucociliary clearance due to immotile cilia, affecting an estimated 1 in 10,000 to 30,000 individuals in the general population [39–41]. LRRC6 is a major PCD-causing gene coding for a 463-amino acid cytoplasmic protein that is exclusively expressed in ciliated tissues, including cells in the respiratory system and testicular cells [42]. Motile cilia are highly complex structures comprising several multiprotein subunits, including ODA and IDA subunits. In our study, the absence of LRRC6 led to a dynein arm defect in cilia. Dynein arm proteins are preassembled in the cytoplasm and are transported to the ciliary axoneme. DNAAF proteins are required for cytoplasmic preassembly of the dynein arm complex [14]. Although existing studies have reported on the role of LRRC6 in dynein arm assembly [16, 42] or trafficking [18], the process with which LRRC6 is involved in ciliogenesis is currently unclear. To gain further insight into the function of LRRC6, we performed RNA-seq analysis in mouse testicular tissues. Notably, in the *Lrrc6* KO group, we identified a downregulation of cilia-related genes, including dynein arm components, strongly suggesting that LRRC6 is not only involved in dynein arm assembly or trafficking but may also govern transcriptional regulation of genes related to the dynein arm assembly or trafficking. LRRC6 has indeed been reported to be involved in the transcriptional regulation of IDAs and ODAs such as DNAI1 and DNAH7 [16]. Similarly, one study reported that PCD patients exhibited a low level of one dynein arm protein, DNAH5, in the cytoplasm of airway cells [43]. However, how *LRRC6* may mediate transcriptional regulation of the ciliary machinery remains elusive.

Additionally, the molecular mechanism by which LRRC6 regulates the transcription of these genes was unknown as LRRC6 is located in the cytoplasm. As inclusive cilia-related genes were downregulated in *Lrrc6* mutant mice (Fig. 3D), we focused on FOXJ1, a master transcription factor of ciliogenesis. FOXJ1 is critical to ciliogenesis by regulating genes that are related to motile cilia [44] [45]. In this study, the relationship between FOXJ1 and LRRC6 was examined using IHC and IF images. Originally, FOXJ1 was detected in the nucleus of epithelial ciliated cells of both human trachea and wild-type mouse trachea. However, in *Lrrc6* KO mice, FOXJ1 was localized to the cytoplasm of airway cells. Different localization patterns of FOXJ1 on the basis of different *LRRC6* genotypes were further established by assessing the expression pattern of FOXJ1 in basal cell organoid. Compared to FOXJ1 localization in wild-type basal cell organoid, in which it was localized to the nucleus, FOXJ1 did not translocate to the nucleus in the *Lrrc6* KO basal cell organoid. Moreover, this pattern of localization was also identified in HeLa cell line. FOXJ1 protein signals were identified in the cytoplasm of HeLa cells singly transfected with FOXJ1 plasmid, whereas FOXJ1 signals were observed in the nuclei of HeLa cells co-transfected with LRRC6 and FOXJ1. The only time FOXJ1 translocated into the nucleus was when LRRC6 was co-transfected, as determined by multiple experiments (Fig. S5B-D). These findings suggest that LRRC6 is required for nuclear translocation of FOXJ1. To the best of our knowledge, this study is the first to report on the LRRC6 transcriptional regulation of cilia-related genes by modulating nuclear translocation of FOXJ1. Using *Lrrc6* KO mice and basal cell organoid system, we specifically showed that LRRC6 enabled the translocation of FOXJ1 into the nucleus to regulate mRNA levels of cilia-related genes including FOXJ1 signature genes. We then treat nuclear transporter inhibitors to block these processes and demonstrated that importin α was necessary for the nuclear translocation of FOXJ1.

Importantly, our study implies that downregulated cilia-related genes resulting from the mislocalization of FOXJ1 could be a pathway linked to the pathogenesis of motile ciliopathy. In this regard, nuclear localization of FOXJ1 could be an additional metric that could be assessed in future clinical diagnosis of PCD. Furthermore, the mislocalization of FOXJ1 might be a novel therapeutic target considering that Gelomyrtol Forte™, a commercially available drug, is known to normalize FOXJ1 expression [46].

A major limitation in the present study is we were unable to reveal how LRRC6 is directly responsible for the nuclear translocation of FOXJ1 based on the data we have collected. Although we proposed that FOXJ1 enters the nucleus through an interaction with importin α, the association between LRRC6 and FOXJ1 is yet to be elucidated. In addition, we were unable to assess whether the mislocalization of FOXJ1 in the absence of LRRC6 also occurs in the human PCD patients. The downregulated mRNA levels of the ciliogenesis-associated markers were also not evaluated in the human PCD patients. Nonetheless, we believe that our findings will inspire ongoing or future studies in elucidating the underlying mechanism of PCD and in the exploration of novel therapeutic strategies.

## Conclusions

In summary, our results suggest that the failure of preassembly or transport of dynein arm components is insufficient to solely explain dynein arm defect underlying the *Lrrc6* KO phenotypes. Instead, the downregulation of both protein and mRNA expression levels of cilia-related genes via the mislocalization of FOXJ1 may constitute the plausible mechanism that underlies *Lrrc6* KO phenotypes. These findings provide a novel insight into the molecular mechanisms for PCD, which may prove useful for future therapeutic interventions.

## Supplementary Information

**Additional file 1: Fig. S1.** **Generation of** ***Lrrc6*** **knockout mice.**  Schematic diagram of the mouse *Lrrc6* targeting allele Lrrc6^tm1eWtsi^. Small arrows show the location of the primers used for PCR. PCR primers and expected PCR products are illustrated. Mouse genotyping by PCR on genomic DNA. Wild type and *Lrrc6* knockout mice were genotyped by three PCR primers. Upper band  and lower band represent KO allele and WT allele, respectively. LacZ staining in the lung and testis tissues of WT and *Lrrc6* KO mice. Each section was counterstained with nuclear fast red staining. X-gal staining in lung sections of *Lrrc6*^+/-^ mice confirms *Lrrc6* expressed in the bronchiole. X-gal staining in testis sections of *Lrrc6*^+/-^ mice confirms *Lrrc6* expressed in spermatocytes to spermatids. *Lrrc6* KO mice grow slower and are smaller than WT siblings. Body weight of WT, heterozygous, and KO mice at postnatal day 10. *** *P* = 0.001; **** *P* < 0.001. Survival plot shows premature death of *Lrrc6* KO mice. Lateral head images show hydrocephalus in *Lrrc6* KO mice. *Lrrc6* KO mice present random left-right asymmetry. Dextrocardia. H, Heart. Asplenia in *Lrrc6* KO mice. K, Kidney; Sp, Spleen; St, Stomach. Low magnificent images of transmission electron microscope images of *Lrrc6* WT and KO mice trachea. The white rectangles depict regions used in Fig.1I-J; Scale bar, 2μm. Longitudinal section images of motile cilia in trachea tissues using transmission electron microscope  show no difference between WT and KO mice.  Raw TEM images used for central microtubule singlet orientation analysis of *Lrrc6* WT and KO  mice trachea, without mark. Scale bar, 500nm. **Fig. S2.** **Summaries of proteomics.** Schematic view of 11-plex tandem mass tag-mass spectroscopy analysis. Testis lysates of five wild type and six *Lrrc6* knockout  mice were labeled by each isotopic amino acid and pooled for mass analysis. The transformation of non-normalized expression distributions  by quantile normalization. turquoise tone, WT samples; orange tone, *Lrrc6* KO samples. Heatmap shows differentially expressed proteins between *Lrrc6 *WT and KO mice. **Fig. S3.** **Transcriptome analysis.** The transformation of raw counts to reads per kilobase of transcript per million mapped reads followed by quantile normalization. A pie chart show mapped read counts per sample before transformation. A box plot represents expression values of normalized RPKM per samples. Principal component analysis showing distinct separation of wild type and *Lrrc6* knockout mice. Turquoise dots, WT; orange dots, KO. Gene ontology analysis of *Lrrc6* KO versus WT shows that 47 gene sets related to cilium are significantly enriched in WT mice. Dots with black boundaries above the dashed line represent cilium-related gene sets with significant p-value. Size of dots, size of gene set; color of dots, origin of gene set. Gene set enrichment analysis plots of *Lrrc6* KO samples over WT samples for motile cilium assemblyand cilium motility, respectively. Decrements of cilium transcripts is statistically significant. Volcano plot representing fold changes of KO over WT and FDR-corrected p-value of each gene expression. Turquoise dots, downregulated genes in KO samples; orange dots, upregulated genes in KO samples; dots with black boundaries, genes included in cilium gene sets. **Fig. S4.** **LRRC6 resides in cytoplasm during basal organoid differentiation.** Illustration of isolation of basal stem cells from a trachea and culture of basal organoids. Cells dissociated from trachea tissue are embedded in Matrigel and cultured in air-liquid-interface condition for 14 days. Basal organoids are completely differentiated, and motile cilia are fully developed at 14th day of the culture. Images of optical microscopy, scanning electron microcopy, and immunofluorescence with confocal microscopy. red, REPTIN; green, acetylated α-tubulin; blue, DAPI. Immunofluorescence of fully differentiated basal organoids shows that DNAI2 and DNAH5 are localized in motile cilia in WT organoids, DNAI2 is detected in the cytoplasm, not in motile cilia, and DNAH5 is not detected in KO organoids, indicating LRRC6 is necessary for proper localization of DNAI2 and DNAH5.  Immunofluorescence was performed at 2-day interval during 7 days of differentiation and confirms that LRRC6 is present in the cytoplasm, not in the nucleus. **Fig. S5.** **Potential nuclear localization signal sequence of FOXJ1.**  Schematic diagram of FOXJ1 with predicted domains and possible non-classical nuclear localization signal sequence. Amino acid sequence from 71 to 125 residues of FOXJ1 coincides with hydrophobic-P -Y -NLS  syntax, φG/A/Sφφ -Xn- [R/H/K]-X2–5-PY. Sequences with boundaries represent residues essential for composing syntax. D, disordered; DBD, DNA binding domain. Cytoplasmic localization of LRRC6 and FOXJ1  was observed in HeLa cells transfected with LRRC6, FOXJ1  plasmid. Scale bars, 50 nm. Percentage of cells in which FOXJ1 translocated to the nucleus as a result of the transfection conditions. Experiments were repeated more than three times independently.

**Additional file 2: Table S1.** Quantification results of basal feet angle variations.

**Additional file 3: Video S1.** Videomicroscopy of brain ventricles from a P14 wild-type mouse.

**Additional file 4: Video S2.** Videomicroscopy of brain ventricles from a P14 Lrrc6-/- mouse.

**Additional file 5: Video S3.** Videomicroscopy of basal cell organoid from a wild-type mouse.

**Additional file 6: Video S4.** Videomicroscopy of basal cell organoid from a Lrrc6-/- mouse.

## Data Availability

The datasets presented in this study can be found in online repositories. The names of the repository/repositories and accession number(s) can be found below: https://www.kobic.re.kr/kona, KRA2200982. The MS proteomics data were deposited to the ProteomeXchange Consortium via the MassIVE partner repository with the data set identifier PXD038887.

## Abbreviations

BP: Biological process
CC: Cellular components
DEGs: Differentially expressed genes
DNAAFs: Dynein axonemal assembly factors
FOXJ1: Forkhead transcription factor 1
GFP: Green fluorescent protein
GO: Gene ontology
GSEA: Gene set enrichment analysis
H&E: Hematoxylin and eosin
IDA: Inner dynein arm
KO: Knockout
LRRs: Leucine-rich repeats
NLS: Nuclear localization signal
ODA: Outer dynein arm
PBS: Phosphate-buffered saline
PCD: Primary ciliary dyskinesia
PFA: Paraformaldehyde
TEM: Transmission electron microscopy
WT: Wild-type
