# Supplementary material for: LRRC6 regulates biogenesis of motile cilia by aiding FOXJ1 translocation into the nucleus
Source: Cell Commun Signal. 2023 Jun 16;21:142. doi: 10.1186/s12964-023-01135-y (PMC10273532; doi:10.1186/s12964-023-01135-y)
Supplement: Supplementary file 2 — Additional file 1: Fig. S1. Generation of Lrrc6 knockout mice. Schematic diagram of the mouse Lrrc6 targeting allele Lrrc6tm1eWtsi. Small arrows show the location of the primers used for PCR. PCR primers and expected PCR products are illustrated. Mouse genotyping by PCR on genomic DNA. Wild type and Lrrc6 knockout mice were genotyped by three PCR primers. Upper band and lower band represent KO allele and WT allele, respectively. LacZ staining in the lung and testis tissues of WT and Lrrc6 KO mice. Each section was counterstained with nuclear fast red staining. X-gal staining in lung sections of Lrrc6+/- mice confirms Lrrc6 expressed in the bronchiole. X-gal staining in testis sections of Lrrc6+/- mice confirms Lrrc6 expressed in spermatocytes to spermatids. Lrrc6 KO mice grow slower and are smaller than WT siblings. Body weight of WT, heterozygous, and KO mice at postnatal day 10. *** P = 0.001; **** P < 0.001. Survival plot shows premature death of Lrrc6 KO mice. Lateral head images show hydrocephalus in Lrrc6 KO mice. Lrrc6 KO mice present random left-right asymmetry. Dextrocardia. H, Heart. Asplenia in Lrrc6 KO mice. K, Kidney; Sp, Spleen; St, Stomach. Low magnificent images of transmission electron microscope images of Lrrc6 WT and KO mice trachea. The white rectangles depict regions used in Fig.1I-J; Scale bar, 2μm. Longitudinal section images of motile cilia in trachea tissues using transmission electron microscope show no difference between WT and KO mice. Raw TEM images used for central microtubule singlet orientation analysis of Lrrc6 WT and KO mice trachea, without mark. Scale bar, 500nm. Fig. S2. Summaries of proteomics. Schematic view of 11-plex tandem mass tag-mass spectroscopy analysis. Testis lysates of five wild type and six Lrrc6 knockout mice were labeled by each isotopic amino acid and pooled for mass analysis. The transformation of non-normalized expression distributions by quantile normalization. turquoise tone, WT samples; orange tone, Lrrc6 KO [file 12964_2023_1135_MOESM1_ESM.docx]

**Additional file 1**


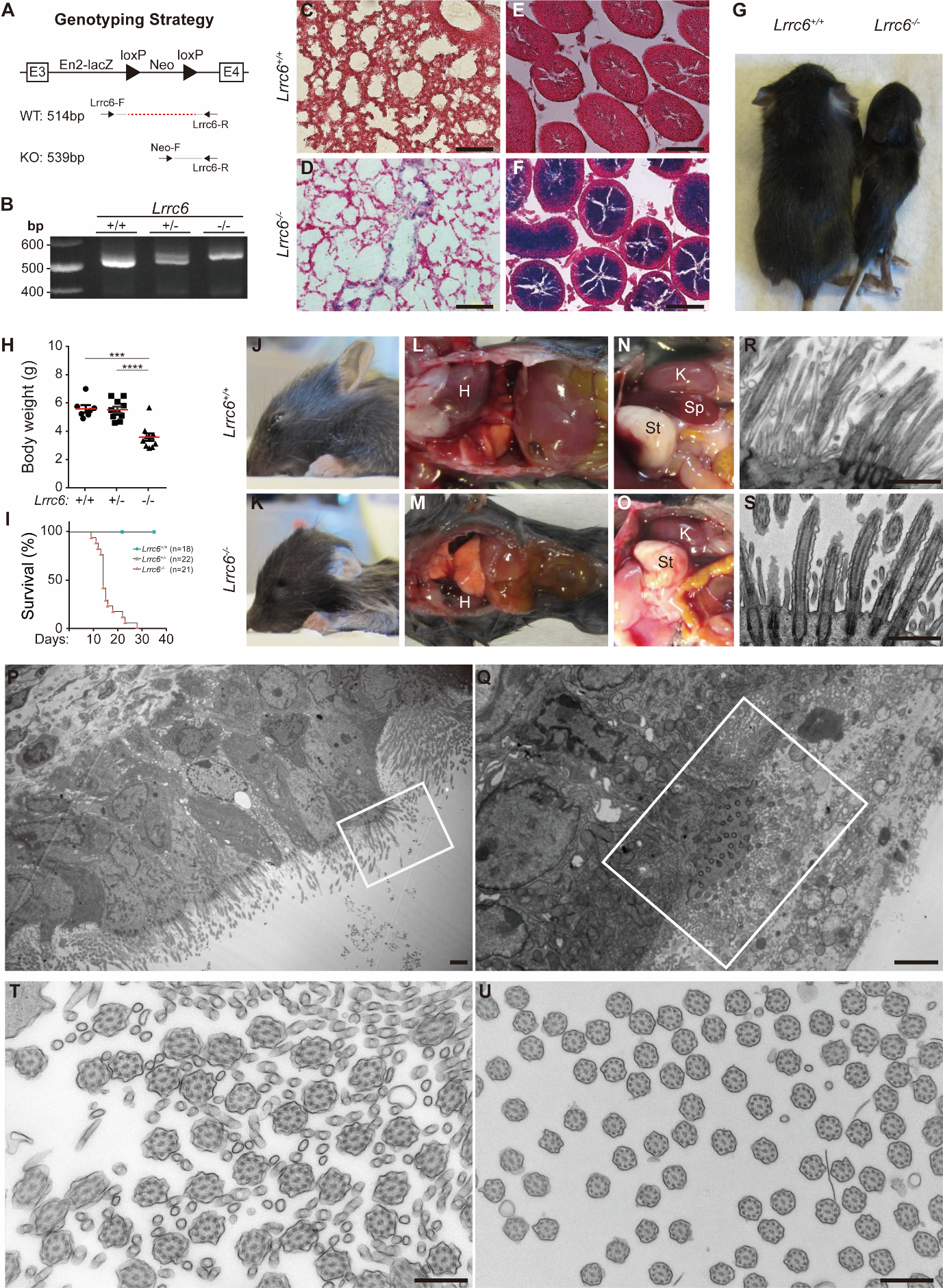


**Fig. S1. Generation of *Lrrc6* knockout mice.** (**A)** Schematic diagram of the mouse *Lrrc6* targeting allele (*Lrrc6*^tm1e(KOMP)Wtsi^). Small arrows show the location of the primers used for PCR. PCR primers (*Lrrc6*-F/*Lrrc6*-R, and Neo-F/*Lrrc6*-R) and expected PCR products are illustrated. **(B)** Mouse genotyping by PCR on genomic DNA. Wild type (WT) and *Lrrc6* knockout (KO) mice were genotyped by three PCR primers (*Lrrc6*-F, Neo-F, and *Lrrc6*-R). Upper band (539 bp) and lower band (514 bp) represent KO allele and WT allele, respectively. (**C-F)** LacZ staining in the lung and testis tissues of WT and *Lrrc6* KO mice. Each section was counterstained with nuclear fast red staining. **(C-D)** X-gal staining in lung sections of *Lrrc6*^+/-^ mice confirms *Lrrc6* expressed in the bronchiole. **(E-F)** X-gal staining in testis sections of *Lrrc6*^+/-^ mice confirms *Lrrc6* expressed in spermatocytes to spermatids. **(G-H)** *Lrrc6* KO mice grow slower and are smaller than WT siblings. **(H)** Body weight of WT (n=7), heterozygous (n=10), and KO (n=10) mice at postnatal day 10. *** *P* = 0.001; **** *P* < 0.001. **(I)** Survival plot shows premature death of Lrrc6 KO mice. (**J-K)** Lateral head images show hydrocephalus in Lrrc6 KO mice. **(L-O)** *Lrrc6* KO mice present random left-right asymmetry. **(L-M)** Dextrocardia. H, Heart. **(N-O)** Asplenia in *Lrrc6* KO mice. K, Kidney; Sp, Spleen; St, Stomach. **(P-Q)** Low magnificent images of transmission electron microscope images of *Lrrc6* WT (P) and KO (Q) mice trachea. The white rectangles depict regions used in Fig.1I-J; Scale bar, 2 μm. **(R-S)** Longitudinal section images of motile cilia in trachea tissues using transmission electron microscope (TEM) show no difference between WT and KO mice. **(T-U)** Raw TEM images used for central microtubule singlet orientation analysis of *Lrrc6* WT (T) and KO (U) mice trachea, without mark. Scale bar, 500 nm.


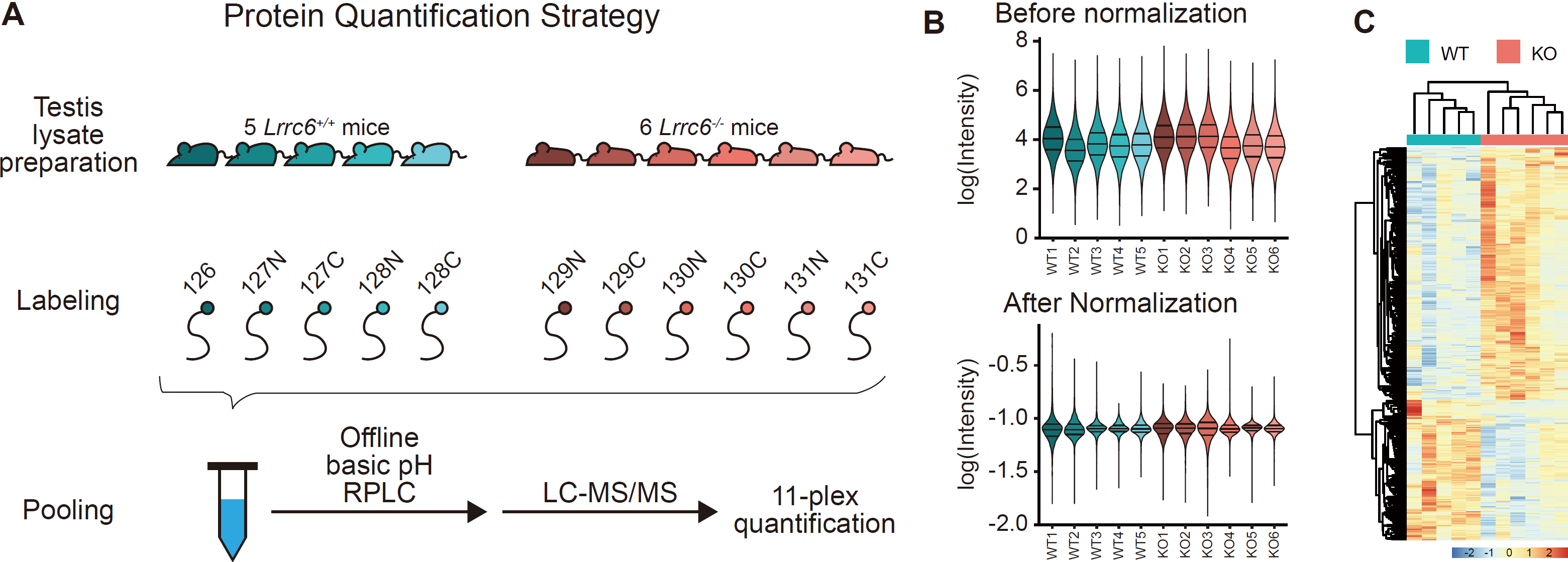


**Fig. S2**. **Summaries of proteomics.** (A) Schematic view of 11-plex tandem mass tag-mass spectroscopy (TMT-MS) analysis. Testis lysates of five wild type (WT) and six *Lrrc6* knockout (KO) mice were labeled by each isotopic amino acid and pooled for mass analysis. **(B)** The transformation of non-normalized expression distributions (upper) by quantile normalization (lower). turquoise tone, WT samples; orange tone, *Lrrc6* KO samples (**C)** Heatmap shows differentially expressed proteins (DEPs) between *Lrrc6* WT and KO mice.


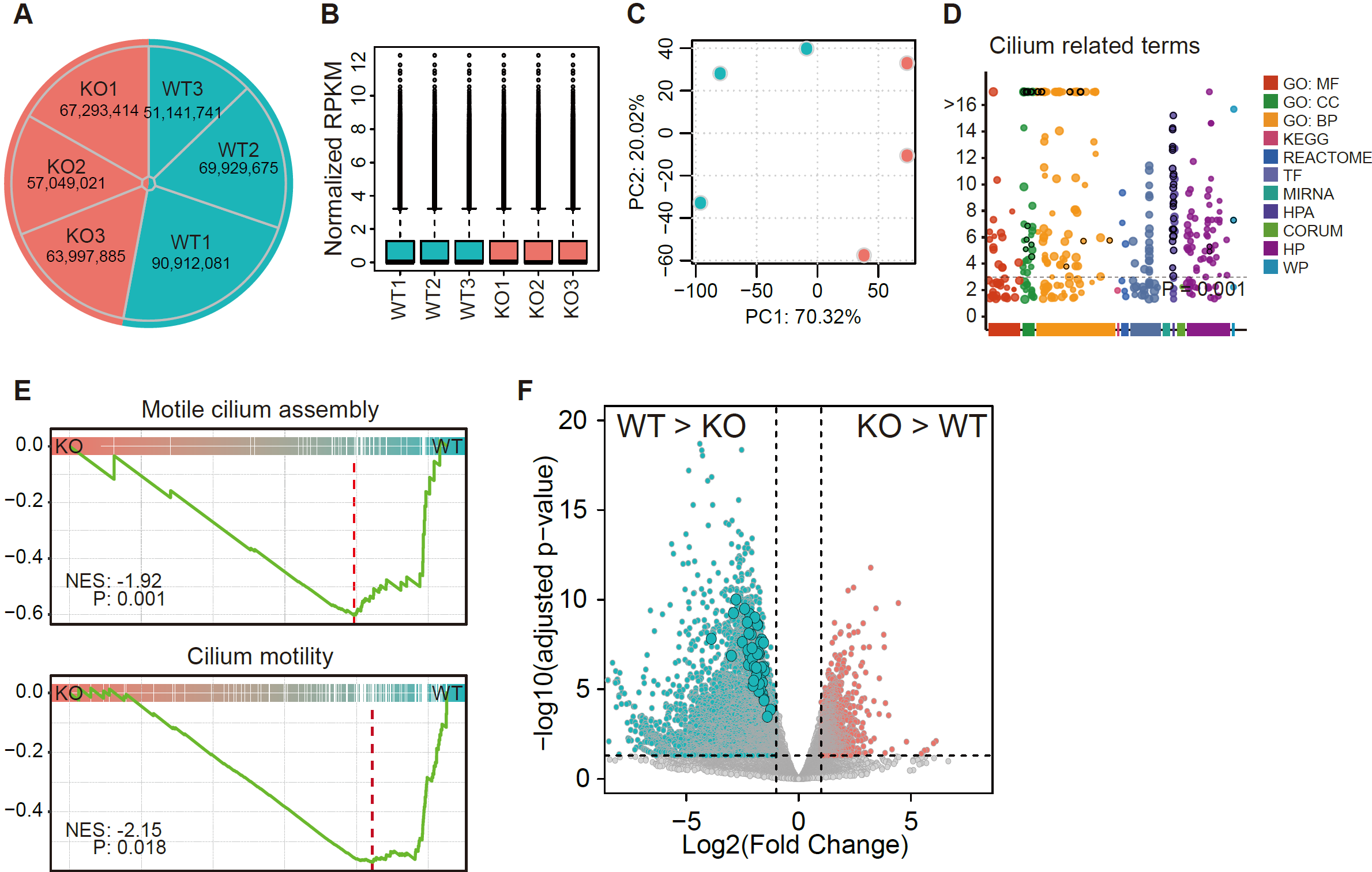


**Fig. S3. Transcriptome analysis.** **(A-B)** The transformation of raw counts to reads per kilobase of transcript per million mapped reads (RPKM) followed by quantile normalization **(A)** A pie chart show mapped read counts per sample before transformation. **(B)** A box plot represents expression values of normalized RPKM per samples. **(C)** Principal component analysis (PCA) showing distinct separation of wild type (WT) and *Lrrc6* knockout (KO) mice. Turquoise dots, WT; orange dots, KO. **(D)** Gene ontology analysis of *Lrrc6* KO versus WT shows that 47 gene sets related to cilium are significantly enriched in WT mice. Dots with black boundaries above the dashed line represent cilium-related gene sets with significant p-value (*P* < 0.001). Size of dots, size of gene set; color of dots, origin of gene set. **(E)** Gene set enrichment analysis (GSEA) plots of *Lrrc6* KO samples over WT samples for motile cilium assembly (upper) and cilium motility (lower), respectively. Decrements of cilium transcripts is statistically significant. **(F)** Volcano plot representing fold changes of KO over WT and FDR-corrected p-value of each gene expression. Turquoise dots, downregulated genes in KO samples; orange dots, upregulated genes in KO samples; dots with black boundaries, genes included in cilium gene sets.


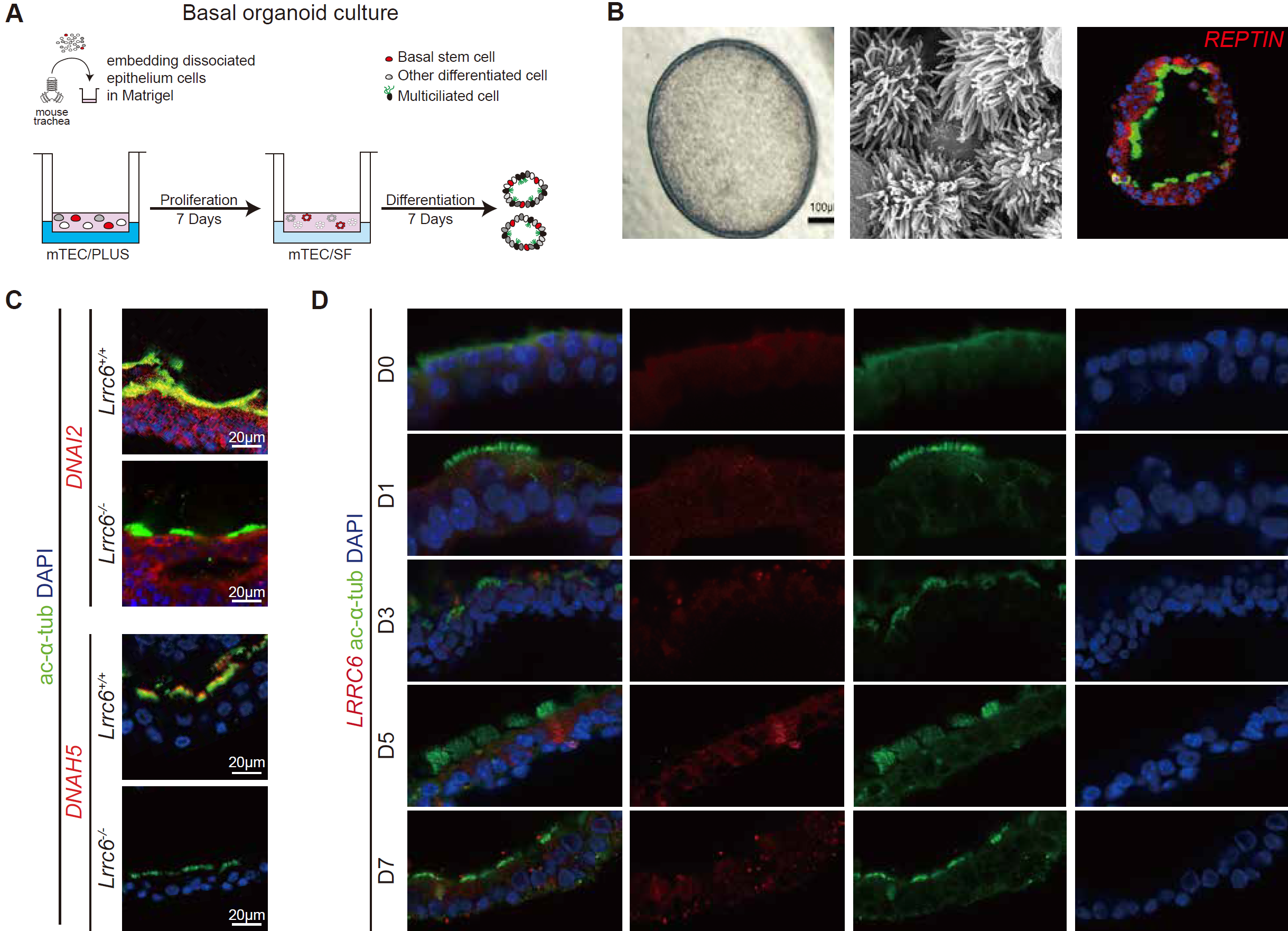


**Fig. S4. LRRC6 resides in cytoplasm during basal organoid differentiation.** **(A)** Illustration of isolation of basal stem cells from a trachea and culture of basal organoids. Cells dissociated from trachea tissue are embedded in Matrigel and cultured in air-liquid-interface condition for 14 days. **(B)** Basal organoids are completely differentiated, and motile cilia are fully developed at 14th day of the culture. Images of optical microscopy (left), scanning electron microcopy (SEM, center), and immunofluorescence with confocal microscopy (right). red, REPTIN; green, acetylated α-tubulin (ac α-tub); blue, DAPI. **(C)** Immunofluorescence of fully differentiated basal organoids shows that DNAI2 and DNAH5 are localized in motile cilia in WT organoids, DNAI2 is detected in the cytoplasm, not in motile cilia, and DNAH5 is not detected in KO organoids, indicating LRRC6 is necessary for proper localization of DNAI2 and DNAH5. **(D)** Immunofluorescence was performed at 2-day interval during 7 days of differentiation and confirms that LRRC6 is present in the cytoplasm, not in the nucleus.


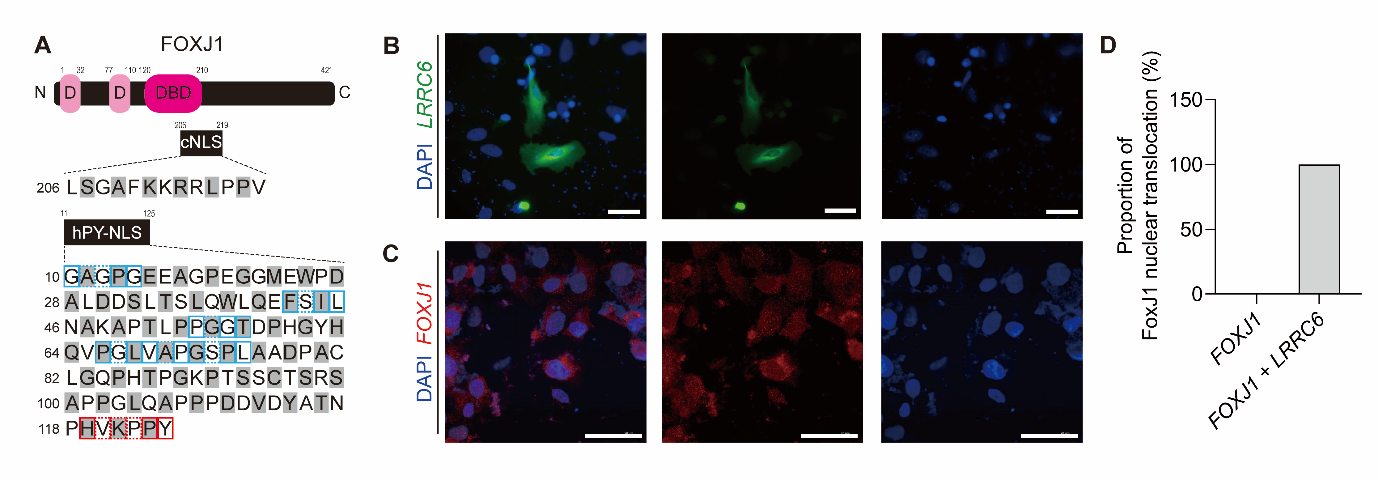


**Fig. S5. Potential nuclear localization signal sequence of FOXJ1.** **(A)** Schematic diagram of FOXJ1 with predicted domains and possible non-classical nuclear localization signal sequence (ncNLS). Amino acid sequence from 71 to 125 residues of FOXJ1 coincides with hydrophobic-P (Proline)-Y (Tyrosine)-NLS (hPY-NLS) syntax, φG/A/Sφφ -Xn- [R/H/K]-X2–5-PY (φ, hydrophobic residue; Xn, any sequence of n residues). Sequences with boundaries represent residues essential for composing syntax. D, disordered; DBD, DNA binding domain. **(B-C)** Cytoplasmic localization of LRRC6 (green) and FOXJ1 (red) was observed in HeLa cells transfected with LRRC6 (B), FOXJ1 (C) plasmid. Scale bars, 50 nm. **(D)** Percentage of cells in which FOXJ1 translocated to the nucleus as a result of the transfection conditions. Experiments were repeated more than three times independently.
