# Supplementary material for: LRRC6 regulates biogenesis of motile cilia by aiding FOXJ1 translocation into the nucleus
Source: Cell Commun Signal. 2023 Jun 16;21:142. doi: 10.1186/s12964-023-01135-y (PMC10273532; doi:10.1186/s12964-023-01135-y)
Supplement: Supplementary file 3 — Additional file 2: Table S1. Quantification results of basal feet angle variations. [file 12964_2023_1135_MOESM2_ESM.docx]

**Table S1. Quantification results of central microtubule singlet angle variations.**

| **Sample** | **Area** | **Mean** | **Min** | **Max** | **Angle** | **Length** |
| --- | --- | --- | --- | --- | --- | --- |
| Mutant | 1.14E-04 | 115.629 | 40 | 211 | -52.927 | 0.113 |
| Mutant | 1.10E-04 | 117.824 | 28 | 228 | -41.285 | 0.11 |
| Mutant | 1.09E-04 | 124.916 | 51 | 233 | -28.951 | 0.108 |
| Mutant | 1.07E-04 | 126.412 | 49 | 211 | -46.548 | 0.105 |
| Mutant | 1.21E-04 | 124.82 | 37 | 219 | -84.193 | 0.119 |
| Mutant | 1.18E-04 | 117.953 | 39 | 183 | -30.466 | 0.119 |
| Mutant | 9.45E-05 | 116.997 | 43 | 203 | -73.887 | 0.094 |
| Mutant | 1.32E-04 | 128.013 | 20 | 216 | 1.79 | 0.129 |
| Mutant | 1.35E-04 | 116.607 | 45 | 219 | -54.866 | 0.133 |
| Mutant | 1.51E-04 | 127.678 | 48 | 226 | -18.677 | 0.15 |
| Mutant | 1.09E-04 | 111.135 | 27 | 193 | -80.362 | 0.108 |
| Mutant | 1.25E-04 | 117.222 | 21 | 218 | -97.472 | 0.124 |
| Mutant | 1.24E-04 | 118.039 | 51 | 178 | -40.4 | 0.124 |
| Mutant | 1.37E-04 | 127.225 | 38 | 219 | -80.676 | 0.136 |
| Mutant | 1.48E-04 | 130.913 | 35 | 234 | -45 | 0.139 |
| Mutant | 1.43E-04 | 125.538 | 51 | 227 | -62.354 | 0.143 |
| Mutant | 1.36E-04 | 123.455 | 44 | 226 | -40.786 | 0.135 |
| Mutant | 1.31E-04 | 122.771 | 40 | 226 | -24.228 | 0.132 |
| Mutant | 1.33E-04 | 121.939 | 49 | 222 | -7.883 | 0.132 |
| Mutant | 1.56E-04 | 127.189 | 32 | 220 | -29.197 | 0.156 |
| Mutant | 1.34E-04 | 115.22 | 41 | 196 | -40.601 | 0.13 |
| Mutant | 1.02E-04 | 95.701 | 12 | 157 | -47.42 | 0.101 |
| Mutant | 1.39E-04 | 123.706 | 6 | 207 | -22.426 | 0.137 |
| Mutant | 1.30E-04 | 110.66 | 35 | 222 | -30.51 | 0.131 |
| Mutant | 1.28E-04 | 122.029 | 22 | 215 | -52.046 | 0.127 |
| Mutant | 1.34E-04 | 111.936 | 25 | 205 | -25.017 | 0.133 |
| Mutant | 1.39E-04 | 107.512 | 43 | 203 | -45 | 0.131 |
| Mutant | 1.19E-04 | 113.283 | 31 | 182 | -54.689 | 0.118 |
| Mutant | 1.40E-04 | 112.461 | 15 | 209 | -32.093 | 0.14 |
| Mutant | 1.09E-04 | 106.1 | 29 | 186 | -61.049 | 0.108 |
| Mutant | 1.12E-04 | 106.031 | 25 | 201 | -56.023 | 0.111 |
| Mutant | 1.11E-04 | 100.342 | 51 | 173 | -37.694 | 0.112 |
| Mutant | 1.01E-04 | 92.474 | 29 | 147 | -60.832 | 0.099 |
| Mutant | 1.17E-04 | 110.214 | 17 | 212 | -13.069 | 0.115 |
| Mutant | 1.07E-04 | 124.52 | 44 | 255 | -23.629 | 0.105 |
| Mutant | 1.19E-04 | 104.828 | 0 | 212 | -8.973 | 0.116 |
| Mutant | 1.12E-04 | 101.375 | 1 | 200 | -11.31 | 0.102 |
| Mutant | 1.11E-04 | 101.435 | 42 | 168 | -36.87 | 0.1 |
| Mutant | 1.18E-04 | 97.791 | 18 | 201 | -41.553 | 0.118 |
| Mutant | 1.06E-04 | 99.466 | 30 | 167 | -56.31 | 0.109 |
| Mutant | 1.15E-04 | 101.523 | 12 | 205 | -9.13 | 0.114 |
| Mutant | 1.04E-04 | 93.324 | 25 | 169 | -58.465 | 0.104 |
| Mutant | 1.09E-04 | 104.483 | 38 | 195 | -45 | 0.102 |
| Mutant | 9.90E-05 | 99.84 | 31 | 157 | -33.69 | 0.101 |
| Mutant | 8.39E-05 | 113.117 | 54 | 172 | -34.077 | 0.082 |
| Mutant | 1.16E-04 | 99.46 | 47 | 169 | -31.477 | 0.115 |
| Mutant | 1.20E-04 | 112.157 | 25 | 214 | -58.707 | 0.12 |
| Mutant | 1.01E-04 | 132.309 | 4 | 255 | -34.992 | 0.098 |
| Mutant | 1.13E-04 | 106.62 | 21 | 211 | -28.856 | 0.112 |
| Mutant | 1.28E-04 | 103.777 | 33 | 170 | -69.228 | 0.125 |
| Mutant | 1.07E-04 | 95.129 | 30 | 183 | -31.891 | 0.106 |
| Mutant | 1.15E-04 | 111.802 | 32 | 205 | -23.86 | 0.114 |
| Mutant | 1.27E-04 | 109.805 | 25 | 204 | -25.741 | 0.125 |
| Mutant | 1.12E-04 | 98.576 | 21 | 179 | -60.642 | 0.111 |
| Mutant | 5.75E-05 | 95.535 | 14 | 171 | -16.504 | 0.057 |
| Mutant | 1.26E-04 | 109.571 | 29 | 214 | -37.794 | 0.125 |
| Mutant | 1.25E-04 | 102.503 | 16 | 197 | -9.31 | 0.124 |
| Mutant | 1.25E-04 | 104.939 | 2 | 192 | -26.98 | 0.124 |
| Mutant | 1.34E-04 | 108.4 | 14 | 213 | -57.995 | 0.133 |
| Mutant | 1.40E-04 | 115.09 | 34 | 226 | -16.858 | 0.138 |
| Mutant | 1.32E-04 | 115.031 | 48 | 215 | -14.47 | 0.129 |
| Mutant | 1.11E-04 | 109.698 | 29 | 188 | 2.121 | 0.109 |
| Mutant | 1.43E-04 | 111.723 | 24 | 212 | -10.67 | 0.141 |
| Mutant | 1.31E-04 | 113.216 | 42 | 244 | 2.726 | 0.127 |
| Mutant | 1.29E-04 | 113.276 | 8 | 196 | 0.909 | 0.127 |
| Mutant | 1.29E-04 | 104.035 | 23 | 220 | -17.301 | 0.128 |
| Mutant | 1.24E-04 | 99.591 | 32 | 164 | -21.448 | 0.121 |
| Mutant | 1.91E-04 | 132.519 | 34 | 222 | -1.828 | 0.189 |
| Mutant | 1.40E-04 | 108.719 | 40 | 219 | -24.305 | 0.137 |
| Mutant | 1.27E-04 | 111.246 | 27 | 197 | 47.603 | 0.125 |
| Mutant | 7.59E-05 | 104.512 | 38 | 183 | 45 | 0.071 |
| Mutant | 1.09E-04 | 102.735 | 37 | 183 | -1.081 | 0.106 |
| Mutant | 9.65E-05 | 99.125 | 24 | 173 | -23.962 | 0.099 |
| Mutant | 1.03E-04 | 97.789 | 3 | 168 | -14.859 | 0.102 |
| Mutant | 9.60E-05 | 104.74 | 12 | 160 | -32.005 | 0.095 |
| Mutant | 1.15E-04 | 106.36 | 30 | 196 | 9.13 | 0.114 |
| Mutant | 1.17E-04 | 106.762 | 34 | 176 | -68.385 | 0.114 |
| Mutant | 1.33E-04 | 106.419 | 23 | 207 | 5.274 | 0.131 |
| Mutant | 1.24E-04 | 95.735 | 15 | 202 | -27.408 | 0.122 |
| Mutant | 1.43E-04 | 113.333 | 31 | 208 | -7.431 | 0.14 |
| Mutant | 1.52E-04 | 122.71 | 38 | 216 | -4.635 | 0.149 |
| Mutant | 1.14E-04 | 109.662 | 0 | 176 | -56.592 | 0.113 |
| Mutant | 1.51E-04 | 118.342 | 21 | 219 | -10.856 | 0.149 |
| WT | 1.01E-04 | 126.797 | 51 | 227 | 14.036 | 0.091 |
| WT | 9.60E-05 | 124.436 | 50 | 200 | 45.855 | 0.095 |
| WT | 1.07E-04 | 121.905 | 35 | 195 | 5.711 | 0.101 |
| WT | 8.85E-05 | 117.39 | 28 | 195 | 28.301 | 0.089 |
| WT | 1.28E-04 | 111.821 | 53 | 218 | 14.036 | 0.116 |
| WT | 1.35E-04 | 119.834 | 30 | 196 | 18.435 | 0.121 |
| WT | 9.60E-05 | 119.667 | 32 | 201 | 32.005 | 0.095 |
| WT | 1.04E-04 | 118.622 | 29 | 189 | 34.624 | 0.102 |
| WT | 1.05E-04 | 108.889 | 42 | 189 | 18.083 | 0.104 |
| WT | 1.07E-04 | 116.629 | 38 | 199 | -8.13 | 0.099 |
| WT | 9.45E-05 | 126.32 | 50 | 221 | 3.731 | 0.093 |
| WT | 1.01E-04 | 115.536 | 33 | 189 | 22.443 | 0.1 |
| WT | 1.15E-04 | 108.226 | 30 | 192 | 21.991 | 0.113 |
| WT | 8.80E-05 | 105.501 | 44 | 165 | 39.289 | 0.086 |
| WT | 8.39E-05 | 109.069 | 50 | 166 | 40.156 | 0.084 |
| WT | 8.19E-05 | 114.255 | 38 | 189 | 55.125 | 0.081 |
| WT | 7.38E-05 | 108.928 | 22 | 202 | 52.815 | 0.073 |
| WT | 1.17E-04 | 121.929 | 53 | 210 | 19.983 | 0.118 |
| WT | 1.00E-04 | 124.809 | 44 | 225 | 32.735 | 0.1 |
| WT | 1.12E-04 | 120.228 | 49 | 212 | 2.121 | 0.109 |
| WT | 1.03E-04 | 113.774 | 32 | 190 | 24.538 | 0.102 |
| WT | 9.60E-05 | 123.648 | 42 | 212 | 40.601 | 0.093 |
| WT | 1.07E-04 | 121.31 | 44 | 179 | -3.302 | 0.105 |
| WT | 1.17E-04 | 97.048 | 42 | 176 | 21.615 | 0.114 |
| WT | 8.19E-05 | 113.185 | 49 | 186 | 30.256 | 0.084 |
| WT | 8.19E-05 | 98.086 | 1 | 202 | 48.013 | 0.081 |
| WT | 1.07E-04 | 133.709 | 56 | 209 | 18.435 | 0.095 |
| WT | 9.15E-05 | 112.43 | 39 | 173 | 14.036 | 0.083 |
| WT | 8.85E-05 | 114.501 | 42 | 209 | 23.025 | 0.087 |
| WT | 8.85E-05 | 119.444 | 44 | 190 | 5.315 | 0.087 |
| WT | 7.94E-05 | 119.419 | 45 | 189 | 21.251 | 0.078 |
| WT | 7.94E-05 | 120.368 | 45 | 192 | 27.897 | 0.077 |
| WT | 7.64E-05 | 119.406 | 48 | 183 | 10.713 | 0.076 |
| WT | 1.04E-04 | 112.557 | 49 | 184 | 33.996 | 0.104 |
| WT | 1.01E-04 | 103.83 | 40 | 166 | -6.981 | 0.099 |
